# Genomic Insights of Pectobacterium carotovorum Strain M022 Quorum-Sensing Activity through Whole-Genome Sequencing

**DOI:** 10.1128/genomeA.01554-14

**Published:** 2015-02-12

**Authors:** Kok-Gan Chan, Wen-Si Tan

**Affiliations:** Division of Genetics and Molecular Biology, Institute of Biological Sciences, Faculty of Science, University of Malaya, Kuala Lumpur, Malaysia

## Abstract

Pectobacterium carotovorum is known to cause serious damage to various major crops worldwide. Here, we report the draft genome of Pectobacterium carotovorum strain M022, a freshwater isolate from a Malaysian waterfall, which has been reported as a plant pathogen and is able to communicate with *N*-acylhomoserine lactone-mediated quorum sensing.

## GENOME ANNOUNCEMENT

Plant pathogens have been a threat to crops and pose a danger to food security. Potato blackleg, which is caused by various soft rot coliforms such as Pectobacterium (1), is one of the major concerns worldwide. Plant pathogenic characteristics, such as production of virulence factors, can be regulated from their communication, which is also known as quorum sensing (QS) (2, 3). QS is the ability of bacteria to express orchestrated physiological activities when a quorate is reached. The plant pathogen, Pectobacterium carotovorum, is a Gram-negative bacteria that destroys various commercially important crops via the production of cell-wall-degrading enzymes (4, 5). In this study, P. carotovorum strain M022 was isolated from a waterfall source. The whole-genome sequencing was conducted for a deeper molecular understanding of this strain.

Initially, a MasterPure DNA purification kit (Epicentre, Inc., Madison, WI, USA) was used to extract the genomic DNA of strain M022 and its quality was checked by a NanoDrop spectrophotometer (Thermo Scientific, Waltham, MA, USA) and a Qubit version 2.0 fluorometer (Life Technologies, Carlsbad, CA, USA). Whole-genome shotgun sequencing of the purified DNA was then performed on an Illumina MiSeq personal sequencer (Illumina Inc., CA, USA), which generated 4,151,785 paired-end reads. Next, the sequences were trimmed and *de novo* assembled with CLC Genomics Workbench version 5.1 (CLC Bio, Denmark), which produced 1,101,735 quality reads (6). The assembly of trimmed reads generated a total of 105 contigs and an *N*_50_ of approximately 126,203.

The draft genome of strain M022 consisted of 4,151,564 bases with an average coverage of 53.3-fold and a G+C content of 51%. Prodigal version 2.60 (7) was used for gene prediction with the prokaryote gene prediction algorithm. In addition, the amounts of tRNAs and rRNAs were predicted using tRNAscanSE version 1.21 (8) and RNAmmer (9), respectively. The strain was then annotated using RAST (10), and 3,600 open reading frames were predicted. In the genomic makeup of strain M022, there are 7 copies of rRNAs and 69 copies of tRNAs.

From the annotation results, the *luxI* and *luxR* homologues of strain M022 were predicted to be located at contigs 33 and 11, respectively. The whole-genome sequence allows deeper understanding of the genetic makeup of P. carotovorum and may help in identifying the link between QS ability with pathogenicity and production of virulence factors (11, 12). It will contribute to an understanding of the mechanism of plant-pathogen interaction and benefit the management of crop disease.

### Nucleotide sequence accession numbers.

This whole-genome shotgun project has been deposited at DDBJ/EMBL/GenBank under the accession number JSXC00000000. The version described in this paper is the first version, JSXC01000000.
